# Hypnosis during Endovascular Abdominal Aortic Aneurysm Repair

**DOI:** 10.3390/jcm13040979

**Published:** 2024-02-08

**Authors:** Lucie Derycke, Quentin De Roux, Nicolas Mongardon, Asmaa Khaled, Marie Corniquet, Pascal Desgranges, Joseph Touma, for the SOS Aorte Paris Est Group

**Affiliations:** 1 Service de Chirurgie Vasculaire, DMU CARE, Assistance Publique-Hôpitaux de Paris (AP-HP), Hôpitaux Universitaires Henri Mondor, F-94010 Créteil, France; lucie.derycke@aphp.fr (L.D.); marie.corniquet@aphp.fr (M.C.); pascal.desgranges@aphp.fr (P.D.); 2 Faculté de Santé, Université Paris Est Créteil, F-94010 Créteil, France; quentin.deroux@aphp.fr (Q.D.R.); nicolas.mongardon@aphp.fr (N.M.); 3 Service d’Anesthésie-Réanimation Chirurgicale, DMU CARE, Assistance Publique-Hôpitaux de Paris (AP-HP), Hôpitaux Universitaires Henri Mondor, F-94010 Créteil, France; asmaakhaled@hotmail.fr; 4 U955-IMRB, Equipe 03 “Pharmacologie et Technologies pour les Maladies Cardiovasculaires (PROTECT)”, Inserm, Université Paris Est Créteil (UPEC), Ecole Nationale Vétérinaire d’Alfort (EnVA), F-94700 Maisons-Alfort, France

**Keywords:** endovascular aortic aneurysm repair (EVAR), hypnosis, non-pharmacological analgesia, anesthesia, hypnotherapy, endovascular surgery

## Abstract

(1) **Background:** Endovascular abdominal aneurysm repair (EVAR) is associated with a reduction in early morbidity and mortality compared with open repair. Procedures performed under hypnosis might represent an alternative to further reduce the risks related to general anesthesia (GA). This study aimed to assess the feasibility and safety of hypnosis and local anesthesia during EVAR. (2) **Methods:** All consecutive patients who underwent EVAR or fenestrated/branched EVAR (f/bEVAR) under hypnosis and local anesthesia (n = 28) between 2017 and 2019 were retrospectively studied and matched to control patients who underwent the same interventions under GA. (3) **Results:** There was neither a significant difference in the length of ICU stay (*p* = 0.06), nor in the occurrence of endoleaks, reintervention, and 30-day mortality rate (*p* = 1.00, 0.73, and 0.24, respectively). The hypnosis group had lower use of norepinephrine (maximum dose 0.04 ± 0.1 vs. 1.2 ± 4.0 mg·h^−1^, *p* < 0.001), shorter procedure duration (181.2 ± 71.4 vs. 214.3 ± 79.6 h, *p* = 0.04), and shorter length of stay (5.4 ± 3.2 vs. 8.4 ± 5.9 days, *p* = 0.002). (4) **Conclusions:** In this pioneering study, hypnosis during EVAR appears feasible and safe. It is associated with lower intraoperative use of norepinephrine, as well as procedure duration and length of in-hospital stay.

## 1. Introduction

Several reports have shown that local anesthesia (LA) with sedation or regional anesthesia (RA) compared with general anesthesia (GA) in endovascular abdominal aneurysm repair (EVAR) decreased cardiac, respiratory, and renal complications. The rate of postoperative admission to the intensive care unit (ICU) and the length of hospitalization were also decreased [1,2,3,4,5,6]. Even though minimally invasive and percutaneous endovascular procedures minimize tissue injury and the need for GA, pain and anxiety still need to be managed in these high-risk patients who could benefit from minimally invasive approaches [7,8]. Intravenous conscious sedation associated to LA may be used, but over-sedation, which can induce cardiovascular depression, hypoxia, or unconsciousness, has to be balanced with the risk of uncontrolled discomfort and restlessness. “Non-pharmacological” analgesia in the form of hypnosis has shown benefits in relieving procedure pain or anxiety [9,10]. It may provide comfort while reducing or even eliminating the need for intravenous sedation. This technique has been used in some surgical procedures with preliminary but promising results [11]. A complex endovascular repair under LA and hypnotherapy case report was previously reported, suggesting its feasibility [12]. Nevertheless, to date, no clinical study has reported experience with hypnosis in endovascular aortic surgery.

The aim of this study was to assess the safety and efficacy of hypnosis associated with LA for patients undergoing EVAR. The primary hypothesis was that hypnosis associated with LA would be non-inferior to GA during EVAR.

## 2. Materials and Methods

### 2.1. Study Design

This manuscript followed the MR004 (Méthodologie de Référence 004) procedure from the National Commission of Data Processing and Freedoms regarding non-interventional studies and was approved by the Henri Mondor Institutional Review Board (IRB 18.11.30.51106 RIPH 2 HPS). The study complied with the principles of the Declaration of Helsinki. According to French law, the need for informed consent was waived due to the retrospective study design. Consecutive patients who underwent EVAR under hypnosis and LA between January 2017 and September 2019 in our institution were retrospectively studied. The hypnotherapy group comprised patients who volunteered for hypnosis after clear information was provided during the pre-anesthesia consultation, delivered by the anesthesiologist. A specific phase for additional information was included in the usual anesthesia consultation and aimed at assessing the patient’s motivation, interests, and family life and providing detailed information on the principle, objectives, and procedure of hypnosis. Exclusion criteria were severe hearing loss, language barrier, and psychiatric disorder. Control patients who underwent EVAR under GA during the same period were retrospectively included and matched using propensity score matching with a ratio of 1:1.5 and accounted for age, cardiovascular diseases, and type of EVAR. All files and data were reviewed by a pair of senior anesthesiologists (QDR and NM) and vascular surgeons (PD and JT).

### 2.2. Intraoperative Features

In the hypnotherapy group, the induction into hypnosis, which consisted of inducing a state of mental focus to bring psychological relief, took about 15 min. The induction technique and the words used were selected depending on the patient’s behavior and reactions. Induction began with fully conscious meditation based on eye fixation, muscle relaxation, slow breathing, and skin sensations. This first phase could last from 5 to 10 min, during which asepsis and draping were performed. The second phase was dissociation. Mental focus on a pleasant life experience or dream was used, inducing a hypnotic trance, to minimize and modify physical pain and/or anxiety. The anesthesiologist monitored the patient’s comfort during the entire procedure. All hypnotherapy procedures were performed by the same anesthesiologist (AK). This practitioner is board certified in medical hypnosis. Residual pain could be relieved with a low dose of opioid analgesic. Norepinephrine infusion was adjusted according to our protocol to a target mean arterial pressure of 65 mmHg, in addition to fluid therapy. Bilateral femoral percutaneous accesses or surgical femoral (+/− axillary) artery exposure were performed under LA (2% lidocaine hydrochloride without epinephrine). A low dose of Propofol or Midazolam was administered at the discretion of the anesthesiologist, according to the patient’s response (based on Richmond Agitation Sedation Scale scores of −1/0). In the GA group, patients underwent intravenous induction and maintenance with either volatile agents or total intravenous anesthesia with a target-controlled infusion of Sufentanil and Propofol and orotracheal intubation. Bispectral index (BIS) values were maintained between 40 and 60 through hypnotic adjustment. 

Surgical procedures were performed using clinically available endografts (Zenith, Cook Medical, Bloomington, IN, USA; Anaconda, Terumo Aortic, Sunrise, FL, USA; Excluder, W. L. Gore & Associates, Flagstaff, AZ, USA; Endurant, Medtronic Vascular, Inc., Minneapolis, MN, USA).

### 2.3. Baseline Characteristics, Follow-Up, and Endpoints

Preoperative patient characteristics (age, gender, body mass index, and co-morbidities) were extracted. EVAR type (infrarenal, fenestrated EVAR (fEVAR), or branched (bEVAR)), type of arterial access, and the clinical classification of the procedure, asymptomatic or symptomatic, were also obtained, according to the Society for Vascular Surgery standards [13]. The primary endpoints were 30-day mortality and reintervention (early, <30 days; and late, >30 days) rates. Secondary endpoints comprised technical success, procedure duration, the use of norepinephrine, intraoperative outcomes, conversion to GA, the lengths of stays in the post-anesthesia care unit (PACU), in the intensive care unit (ICU) and in the hospital, and the occurrence of endoleaks. Procedure duration included anesthesia monitoring, hypnosis or GA induction, endovascular aortic repair, and recovery times until departing the post-anesthesia care unit. Technical success and intra- and postoperative outcomes were defined according to the Society for Vascular Surgery standards [13]. Primary technical success was defined as the successful introduction and deployment of the device in the absence of surgical conversion or mortality, type I or III endoleaks, or graft limb obstruction. Secondary technical success was defined as if unplanned endovascular or surgical procedures were required to achieve technical success.

### 2.4. Statistical Analysis

The continuous variables are presented as means ± standard deviations. We performed propensity score matching with 1:1.5 nearest neighbor matching using a logistic regression. The defined matching criteria were age, sex, body mass index, ASA score of III–IV, diabetes, chronic kidney disease, coronaropathy, hypertension, stroke, smoking, timing of surgery, and EVAR type. The balance between the two matched groups was evaluated by the standardized mean differences in the matching variables. After propensity score matching, the between-group differences were compared using unpaired Student’s t-tests or Mann–Whitney U tests. The categorical variables are presented as frequencies (with percentages) and the between-group differences were compared using a Chi-square test. *p* < 0.05 was considered statistically significant. The analysis was performed using R statistical software version 4.2.2 (a language and environment for statistical computing; R Foundation for Statistical Computing, Vienna, Austria). 

## 3. Results

Twenty-eight patients were treated by EVAR or f/bEVAR with hypnosis during the study period. Forty-three patients who underwent EVAR or f/bEVAR during the same period were matched and included in the control group. Secondary technical success was 100% in both groups.

The baseline demographic and clinical characteristics of the patients in each group are presented in Table 1. All cases of symptomatic aneurysm had pain without aneurysmal rupture and were treated within one day.

### EVAR Characteristics and Outcomes

The procedural characteristics and outcomes are presented in Table 2. The distribution of EVAR type (simple or complex) was similar between the two groups (hypnosis vs. GA: 28.6% vs. 32.6%, *p* = 0.93). A significant difference was calculated in the percutaneous approach rate with a significantly higher rate of percutaneous approach in the hypnotherapy group (hypnosis vs. GA: 75% vs. 46.5%, *p* = 0.03). Three patients underwent EVAR for urgent AAA, one in the hypnotherapy group and two in the GA group (hypnosis vs. GA: 3.6% vs. 4.7%, *p* = 0.99).

Significant differences were observed in procedure duration (hypnosis vs. GA: 181.2 ± 71.4 min vs. 214.3 ± 79.6 min, *p* = 0.04) and intraoperative maximum dose of norepinephrine (hypnosis vs. GA: 0.04 ± 0.1 mg·h^−1^ vs. 1.2 ± 4.0 mg·h^−1^, *p* < 0.001). Arterial percutaneous access or surgical artery exposure of all patients in the hypnosis group were performed under LA. A proportion of 53.6% of patients in the hypnosis group (15/28) required a low dose of hypnotic drug administration (Propofol (12/15) or Midazolam (3/15)) and 78.6% (22/28) required low dose of opioid analgesic (Sufentanil).

No statistically significant difference in the intraoperative complication rate was calculated between study groups. Causes of intraoperative complications are reported in Table 3. 

Conversion to GA was required in two (8%) patients who underwent EVAR in the hypnotherapy group for major discomfort. No further significant differences were found regarding the length of stay in the PACU and the ICU, the endoleaks occurrence, and the reintervention rates. Neither aspiration pneumonia nor anesthesia-related complications were noted. None of the patients reported perioperative memory or awareness. Several complications were observed in both groups. Causes of early reintervention are reported in Table 4. Medically severe outcomes were cardiac decompensation (2), resolutive renal failure (2), myocardial ischemia (1), femoral hematoma (1), and prostatitis (2). None of them had an apparent relation with anesthetic management. A significant difference was observed in terms of in-hospital length of stay (hypnosis vs. GA: 5.4 ± 3.2 days vs. 8.4 ± 5.9 days, *p* = 0.002). There were three early deaths in the GA group, which occurred as a result of intraoperative iliac rupture, severe postoperative ischemia colitis and postoperative myocardial ischemia. Two of them occurred after complex EVAR repair. No statistically significant difference in the 30-day mortality rate was calculated between study groups. 

## 4. Discussion

The results of this study suggest that use of hypnosis with LA during EVAR or b/fEVAR is feasible and comparable to GA in terms of safety. An association between hypnosis with LA and a faster recovery and greater intraoperative hemodynamic stability was noticed when compared to GA. Thirty minutes of procedure time were saved in the hypnosis group in our study compared with standard care, despite the time invested in the hypnotic induction, as it can be performed outside the operating room in the pre-anesthesia room.

These encouraging and pioneering results should be confirmed by larger studies that compare hypnosis and LA to sedation and LA. Indeed, minimally invasive and percutaneous endovascular procedures, such as EVAR, minimize tissue injury and the need for GA [14]. No study has reported results on hypnosis compared to GA in endovascular aortic surgery, but several studies have shown a benefit of conscious sedation and LA in EVAR [1,3,4,5,6,15,16]. Verhoeven et al. published a prospective cohort study of 239 patients and demonstrated an overall lower incidence of complications in the local and regional anesthesia groups than in the GA group [1]. Moreover, the procedure time and length of stay in the ICU were shorter in the LA group, as well as the length of stay in hospital, the time to ambulation, and the time to resuming a regular diet. Data from the EUROSTAR registry showed in particular that high-risk patients benefit from less invasive anesthetic techniques [15,17]. Bross et al., in 2015, examined outcomes of EVAR in 1261 patients and highlighted that the use of LA or regional anesthesia appeared to reduce operative duration, ICU admission, and length of postoperative hospital stay [16]. Fewer pulmonary complications were also reported in the LA subgroup of a large analysis of 8141 patients [6]. Finally, Armstrong et al. published a systematic review of the anesthetic strategy impact on post-EVAR outcomes [2]. The twenty-two analyzed studies were observational or consisted of a secondary analysis of trial data. Results suggested that the type of anesthesia may be associated with improved outcomes, both for emergency and elective EVAR. Particularly, LA seems to have a positive effect on complication after emergency EVAR. However, to manage pain and anxiety, intravenous conscious sedation is frequently used in combination with LA. The anesthesiologist has to balance the risk of over-sedation, which can induce cardiovascular depression, hypoxia, or unconsciousness, and the risk of uncontrolled discomfort and restlessness [18,19]. Endovascular procedures are increasingly performed under LA ± light sedation, including simple endovascular aortic procedures. The safety and feasibility of hypnosis adjunctive to LA ± light sedation was evaluated in this pioneering study in comparison with our standard practice. But our local experience is also evolving in this direction. Larger studies focused on hypnosis with LA ± light sedation compared to LA ± light sedation should be the next step to draw conclusions about the benefits of hypnosis in EVAR.

Hypnosis may therefore represent a potential tool in addition to LA to reduce the intraoperative need for analgesic and hypnotic drugs, without additional cost or specific adverse effects. Its mechanisms are not yet well understood; some researchers report that hypnosis is related to an altered state of consciousness, while other theories describe psychological concepts such as clinician–patient expectations. “Non-pharmacological” analgesia in the form of hypnosis has demonstrated a positive effect on pain and anxiety relief, with a reduced use of analgesic and anti-anxiety medication [9]. Several observational studies and meta-analyses have reported its benefits on postoperative outcomes and the duration of stay in the ICU [20,21,22,23,24,25,26,27,28]. Hypnosis can be used preoperatively or intraoperatively with hypno-sedation. In a prospective and randomized study in thyroid surgery, Defechereux et al. showed the safety of hypnosis and reported significant postoperative positive outcomes [29]. More recently, a retrospective study focused on patients’ comfort showed good overall patient satisfaction [26]. Several studies have also reported favorable results in breast surgery. Berliere et al. showed beneficial effects of hypnosis on anxiety, postoperative asthenia, pain, length of hospital stay, and the incidence of side effects of chemotherapy and radiotherapy [27]. In addition, the HYPNOSEIN randomized clinical trial focused on preoperative hypnosis before GA during minor breast cancer surgery and reported benefits regarding PACU length of stay, asthenia, anxiety, and patient satisfaction [28]. The most recent meta-analysis analyzed 50 randomized studies and found an effect in favor of hypnosis in various outcomes relevant to surgery, such as mental distress, pain, medication consumption, recovery, and surgical procedure time [25]. Our experience suggests similar results in endovascular aortic surgery. Indeed, in our retrospective study, an association between hypnosis and LA and a lower use of norepinephrine, shorter length of procedure duration, and length of hospital stay was observed when compared to GA. Shorter operating times could be explained by several factors. First, the induction stage of hypnosis is fairly rapid if the patient is well prepared and takes no longer than GA induction, as is the hypnotic trance emergence stage. Second, the involvement of the entire team is required to reduce acoustic stimulation, which could help the medical team to focus and ensure the smooth running of the surgical procedure. Nevertheless, the difference in the proportion of percutaneous femoral approach use between the groups may have impacted the variable. Finally, one study showed that hypnosis in radiologic procedures has the potential to be cost-effective when compared to standard intravenous conscious sedation [9].

The catecholamine-sparing nature of this strategy may be a potential advantage for high-risk patients. Hypnosis may provide comfort while reducing or even eliminating the need for intravenous sedation. The state of trance induced by hypnosis alters perioperative pain sensation and allows immobility and communication during the procedure. Hypnosis is promising for EVAR as the patient remains cooperative during the procedure, whereas confusion may be observed with conscious sedation, preventing the use of image fusion techniques. These results are also promising for the use of hypnosis in addition to LA in complex EVARs with long procedure durations for high-risk patients deemed unfit for GA. Indeed, complex EVARs, which require strict immobility of the patient for a long period of time, are almost always performed under GA. However, a patient’s participation in hypnosis requires a full understanding of the process. Patients with psychotic or cognitive disorders cannot be eligible.

This pilot cohort is, to the best of our knowledge, the first report of major vascular surgery performed under hypnosis. Only a case report from our group has been published in this setting [7]. Even if most of the procedures were scheduled surgery, a few procedures were emergent. This underlines the feasibility of the technique in selected patients. 

However, our study has several limitations. It is a retrospective work, and the number of subjects was small because of the single-center design. Therefore, statistical analysis cannot allow conclusions to be drawn about outcomes and causes of reintervention. Moreover, the patient’s pain or anxiety, as well as the patient’s experience and satisfaction, were not evaluated. Therefore, conclusions about the positive effect of hypnosis on the outcomes cannot be firmly drawn, as these elements would be essential to evaluate. Selection bias may not be entirely excluded (e.g., percutaneous or surgical femoral approach, which may have an impact on the length of procedure and hospital stay). Patients susceptible to benefit from hypnosis were preoperatively selected on a voluntary basis and may represent a particular type of participant. Indeed, only voluntary patients received hypno-sedation. Nevertheless, this method of patient selection reflects clinical practice. Indeed, to be effective, hypnosis requires full participation and understanding, and an uncooperative patient will not be receptive. Conversely, due to the retrospective design, we compared here several combinations of sedation regimens, precluding firm conclusions. In addition, patients under LA + hypnosis should be compared with patients under only LA to further investigate the specific impact of hypnosis. Importantly, a large portion of patients under hypnosis required additional I.V. sedation or analgesia, in complement to LA. Only a minority of procedures could be performed under strict hypnosis and LA. Notably, our hospital LOS was relatively long in both groups, due to the comorbidities of the patients from our referral tertiary center and the inclusion of complex cases. Despite these limitations, hypnotherapy in EVAR does not seem to negatively impact clinical outcomes. This pioneering study showed the feasibility and safety of hypno-anesthesia during endovascular aortic surgery and its benefits will need to be demonstrated in a larger report.

## 5. Conclusions

Even in complex cases, hypnotherapy in EVAR does not seem to negatively impact clinical outcomes. In this study, associations between hypnosis and LA and a reduction in the intraoperative use of norepinephrine, procedure duration, and length of hospital stay were seen when compared to GA. Nevertheless, prospective and randomized studies are needed to identify the psychological and clinical benefits of hypnosis in aortic endovascular procedures.

## Figures and Tables

**Table 1:** Baseline demographic and clinical characteristics of the patients of the EVAR and f/bEVAR groups.

**Variable**	**EVAR Group**		***p* Value**
	**Hypnosis (n = 20)**	**GA (n = 29)**	
Age	70.2 ± 10.5	74.9 ± 8.1	0.06
Male	20 (100)	26 (89.7)	0.38
ASA = 3 or 4	14 (65)	19 (66)	0.67
Diabetes	5 (25)	3 (10.3)	0.33
Chronic kidney disease	4 (20)	5 (17.2)	0.99
Coronaropathy	7 (35)	13 (44.8)	0.69
Hypertension	17 (85)	21 (72.4)	0.49
History of stroke	1 (5)	2 (6.9)	0.99
BMI (kg/m^2^)	26.8 ± 2.7	26.4 ± 3.8	0.47
Smoker	5 (25)	12 (41.4)	0.38
Symptomatic aneurysm	1 (5)	2 (6.9)	0.99
	**f/bEVAR Group**		
	**Hypnosis (n = 8)**	**GA (n = 14)**	
Age (yo)	72.6 ± 9.6	75.2 ± 11.4	0.71
Male	8 (100)	12 (85.7)	0.73
ASA = 3 or 4	6 (75)	10 (71)	0.59
Diabetes	0 (0)	1 (7.1)	0.99
Chronic kidney disease	0 (0)	2 (14.3)	0.73
Coronaropathy	3 (37.5)	7 (50)	0.90
Hypertension	8 (100)	8 (100)	0.20
History of stroke	1 (12.5)	2 (14.3)	0.99
BMI (kg/m^2^)	26.5 ± 3.6	26.4 ± 4.2	0.78
Smoker	4 (50)	2 (14.3)	0.19
Symptomatic aneurysm	0 (0)	0 (0)	0.20

Data are presented as mean ± standard deviation or no. (%). ASA: American Society of Anesthesiologists; BMI: body mass index; EVAR: endovascular abdominal aneurysm repair; GA: general anesthesia.

**Table 2:** Procedural characteristics and outcomes.

Variable	Hypnosis (n = 28)	GA (n = 43)	*p* Value
Percutaneous access	21 (75)	20 (46.5)	**0.03**
Procedure duration (min)	181.2 ± 71.4	214.3 ± 79.6	**0.04**
In-hospital length of stay (day)	5.4 ± 3.2	8.4 ± 5.9	**0.002**
ICU length of stay (day)	0.1 ± 0.8	0.7 ± 1.7	0.06
PACU length of stay (h)	9.0 ± 6.4	9.1 ± 6.0	0.95
Red blood cell transfusion (units)	0.3 ± 0.8	0.8 ± 1.9	0.32
Maximum norepinephrine use (mg/h)	0.04 ± 0.1	1.2 ± 4.0	**<0.001**
Intraoperative complication	2 (7.1)	6 (14.0)	0.61
Type I endoleak	2 (7.1)	2 (4.7)	0.99
Early reintervention	3 (10.7)	7 (16.3)	0.73
Late reintervention	0 (0)	2 (4.7)	0.52
30-day mortality	0 (0)	3 (7.0)	0.24

Data are presented as mean ± standard deviation or no. (%). ICU: intensive care unit, GA: general anesthesia; PACU: post-anesthesia care unit. Significant *p* values are represented in bold.

**Table 3:** Intraoperative complications.

Outcome	Hypnosis (n = 28)	GA (n = 43)	Total	*p* Value
Failure of sheath navigation	1	0	1	0.83
Partial coverage of renal artery	1	2	3	0.99
Endograft eg thrombosis	0	1	1	0.99
Failure of target artery catheterization	0	1	1	0.99
Target artery thrombosis	0	1	1	0.99
Iliac rupture	0	1	1	0.99
Total	2 (7.1)	6 (14)	8 (11.3)	0.62

Data are presented as no. (%). GA: general anesthesia.

**Table 4:** Causes of early reintervention.

Cause	Hypnosis (n = 28)	GA (n = 43)	Total	*p* Value
Stentgraft kink	1	0	1	0.83
Type I endoleak	2	2	4	0.99
Acute peripheral ischemia	0	1	1	0.99
Endograft leg thrombosis	0	2	2	0.67
Femoral false aneurysm	0	1	1	0.99
Severe postoperative ischemia colitis	0	1	1	0.99
Total	3 (10.7)	7 (16.3)	10 (14.1)	0.73

Data are presented as no. (%). GA: general anesthesia.

## Data Availability

Data are contained within the article.
